# Assessing the relationships between emotion regulation, depression, anxiety, and stress symptoms in a Tunisian University Setting

**DOI:** 10.1192/j.eurpsy.2024.1113

**Published:** 2024-08-27

**Authors:** S. Boudriga, M. Lagha, M. Methni, Y. Ben Youssef, I. Ben Romdhane, W. Homri, R. Labbane

**Affiliations:** Psychiatry C, Razi Hospital, Manouba, Tunisia

## Abstract

**Introduction:**

Difficulties in emotion regulation are known to be associated with various mental health problems, particularly depression, and anxiety.

**Objectives:**

This study aimed to explore the relationship between emotion regulation, depression, anxiety, and stress symptoms in a Tunisian university setting.

**Methods:**

A cross-sectional, descriptive, and analytical study was conducted among Tunisian students from August to September 2023. Data were collected through an anonymous online questionnaire, assessing sociodemographic characteristics, the Arabic version of the difficulties in emotion regulation scale short form (DRES-SF), and the depression, anxiety, and stress scale (DASS-21).

**Results:**

A sample of 307 university students were enrolled. The sex ratio (M/F) was 0.28. In the assessment of emotional regulation difficulties, participants reported a mean total score of 42.47 ± 12.68. The mean score of stress was 18.2 ± 11.36, reflecting a mild severity. Participants with higher stress levels have more difficulties in emotional regulation (r=0.658, p=0.00). Participants with depressive symptoms showed a higher DRES-SF total score (r= 0.629, p=0.00). Participants had a mean anxiety score of 15.6 ± 10.57, reflecting a severe severity. A significant correlation between total DRES-SF score and anxiety (r=0.606, p=0.00).

**Conclusions:**

Our study concluded significant positive correlations between depressive symptoms, anxiety, and stress symptoms with emotion regulation difficulties.

**Disclosure of Interest:**

None Declared

